# Poor Prognosis of Gastric Adenocarcinoma with Decreased Expression of AHRR

**DOI:** 10.1371/journal.pone.0043555

**Published:** 2012-08-27

**Authors:** Yuan-fang Li, Dan-dan Wang, Bai-wei Zhao, Wei Wang, Shu-qiang Yuan, Chun-yu Huang, Yong-ming Chen, Yan Zheng, Rajiv Prasad Keshari, Jian-chuan Xia, Zhi-wei Zhou

**Affiliations:** 1 State Key Laboratory of Oncology in South China and Department of Experimental Research, Sun Yat-sen University Cancer Center, Guangzhou, People’s Republic of China; 2 Department of Gastric and Pancreatic Surgery, Sun Yat-sen University Cancer Center, Guangzhou, People’s Republic of China; National Cancer Center, Japan

## Abstract

**Background:**

The aryl hydrocarbon receptor (AHR) repressor (AHRR), a member of growing superfamily, is a basic-helix-loop-helix/Per-AHR nuclear translocator (ARNT)-Sim (bHLH-PAS) protein. Recently, AHRR has been proposed to function as a putative new tumor suppressor gene based on some relevant studies in multiple types of human cancers. This current study aims to investigate AHHR expression and its prognostic significance in primary gastric adenocarcinoma.

**Methodology/Principal Findings:**

The expression level of AHRR was analyzed using real-time quantitative PCR (RT-qPCR), western blotting, and immunohistochemical staining. It was clearly showed that the expression status of AHRR was reduced in tumor tissue samples compared with that in matched adjacent non-tumor tissue samples by RT-qPCR (*P* = 0.0423) and western blotting analysis (*P* = 0.004). Moreover, data revealed that AHRR without exon 8 (the active isoform) was the predominant form either in tumor tissues (66.7%, 8/12) or in matched adjacent non-tumor tissues (100.0%, 12/12), and the mRNA level of this isoform was significantly reduced in tumor tissues (*P* = 0.006). Immunohistochemistry analysis indicated that AHRR expression was significantly decreased in 175 of 410 (42.7%) gastric adenocarcinoma cases. Kaplan-Meier survival curves and Multivariate Cox analysis revealed that decreased expression of AHRR was significantly associated with poor prognosis in gastric adenocarcinoma patients.

**Conclusions/Significance:**

Our data suggests that, in primary gastric adenocarcinoma, AHRR may play as a suppressor gene and its expression status has the potential to be an independent prognostic factor.

## Introduction

Gastric cancer, with an estimated number of one million new cases every year [1], is the fourth most common malignant tumor worldwide and the second most common cause of cancer-related deaths each year (10.4% of cancer deaths) [2]. Gastric cancer treatment consists of a combination of surgery, chemotherapy, and radiation therapy. However, nearly 60% of patients succumb to gastric cancer even after curative resection alone or after adjuvant therapy [3]. Gastric cancer is a heterogeneous disease in both histology and genetics; hence, the outcome of patients is difficult to predict with classical histological classifications. Tumor progression is considered to be a multifactorial and multistep process that involves the activation of oncogenes and the inactivation of tumor suppressor genes at different stages. Recently, several new oncogenes and tumor suppressor genes associated with gastric cancer have been confirmed, which may be useful for early diagnosis and the development of molecularly targeted therapies [4], [5]. To improve the prognosis of gastric adenocarcinoma, further understanding of the molecular mechanisms of cancer progression and the development of new therapeutic tools based on these mechanisms are anticipated [4], [6]–[8].

AHRR, a bHLH-PAS transcription factor, is located in chromosome 5p15.3 that has been proposed to contain one or more tumor suppressor genes [9]. The protein encoded by this gene participates in the AHR signaling cascade, which mediates the toxic effects of dioxin, including teratogenesis, immunosuppression and tumor promotion, and is involved in the regulation of cell growth and differentiation [10]–[12]. An inhibitory activity of AHRR on AHR signaling was proposed from overexpression studies [11]. In cellular systems, Mimura *et al* reported a gene dose dependent repressive effect of the AHRR and this activity is selectively due to displacement or inhibition of AHR binding to XREs (xenobiotic-responsive element) [13]. Besides, Evans *et al* reported a similar effect of AHRR on AHR signaling by transient transfection assays with zebrafish AHRRs [14].

AHRR gene encodes two isoforms and the isoform without exon 8 has been reported to be the active isoform, which is the predominant form of AHRR expressed in multiple human tissues and human tumor cell lines [15]. Karchner *et al* reported that AHRR isoform lacks exon 8 formed a complex with AHR nuclear translocator (ARNT), and this isoform did not repress the nuclear receptor pregnane X receptor or estrogen receptor, but did repress HIF (hypoxia-inducible factor)-dependent signaling [15].

Recently, lost or reduced expression of AHRR has been observed in many types of cancerous human tissue [16], including hepatocellular carcinoma, colon carcinoma, prostate cancer, breast cancer and others [9], [17], [18], demonstrating that AHRR is a putative new tumor suppressor gene in multiple types of human cancers [9]. However, to the best of our knowledge, no previous reports exist concerning the expression status of AHRR and the prognostic value of this protein in primary gastric adenocarcinoma. In this study, the expression of AHRR in primary gastric adenocarcinoma was estimated using quantitative real-time PCR (qRT-PCR), western blotting and immunohistochemistry. Additionally, we identified the relationship between AHRR expression and clinicopathological features, and we evaluated its prognostic value to post-resection survival in gastric cancer.

## Results

### AHRR mRNA Expression Analyzed with qRT-PCR

**Figure 1:**
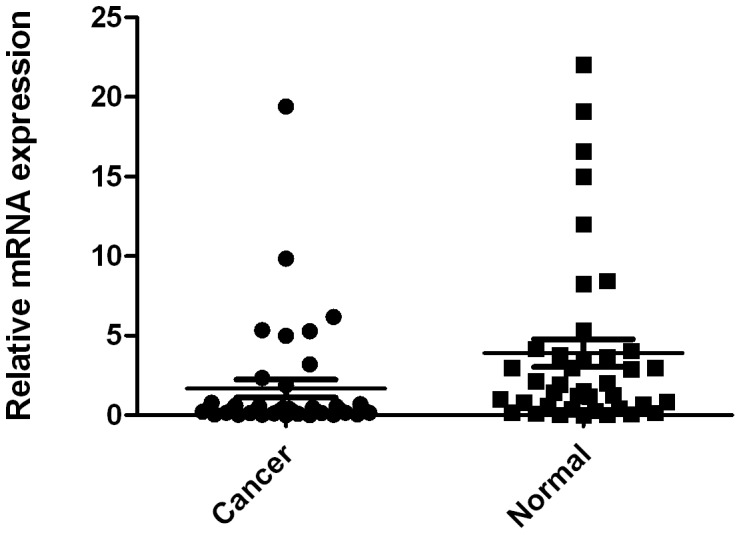
Decreased mRNA expression of AHRR in gastric cancer tissues as assessed by real time quantitative RT-PCR (n = 40, *P* = 0.0423). The horizontal lines represent the means.

The mRNA levels of AHRR were estimated by qRT-PCR assays on 40 pairs of resected specimens (tumor tissue samples and matched adjacent non-tumor tissue samples) from eligible gastric cancer patients. The AHRR mRNA levels were significantly reduced in 27 (67.5%) tumor tissue samples, compared with the adjacent non-tumor tissue samples (*P* = 0.0423, Figure 1).

### mRNA Expression of Two AHRR Isoforms Analyzed with qRT-PCR

**Figure 2:**
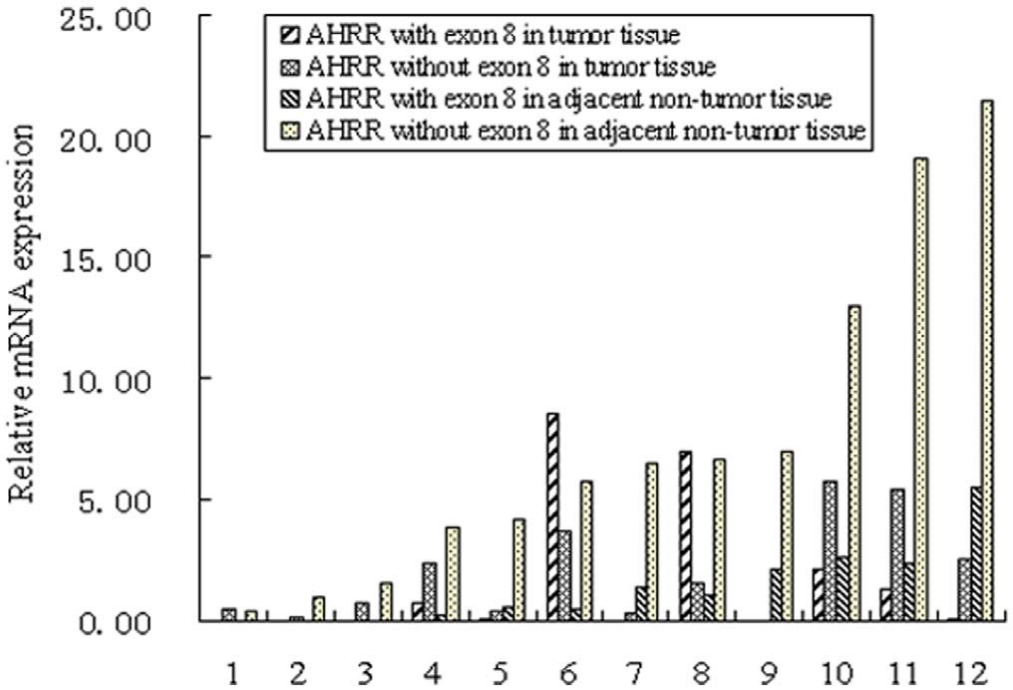
Relative mRNA expression of two AHRR isoforms analyzed by qRT-PCR (n = 12). The total AHRR mRNA expression and the AHRR isoform without exon 8 expression were significantly reduced in tumor tissues compared with the adjacent non-tumor tissues (*P* = 0.045 and 0.006, respectively). The AHRR isoform without exon 8 was the predominant form either in tumor tissues (66.7%, 8/12) or in matched adjacent non-tumor tissues (100.0%, 12/12).

The relative mRNA expression of two AHRR isoforms (with or without exon 8) was determined by qRT-PCR on 12 cases of resected specimens (tumor tissue samples and matched adjacent non-tumor tissue samples) from eligible gastric cancer patients. Data revealed that the total AHRR mRNA expression and the AHRR isoform without exon 8 expression were significantly reduced in tumor tissues compared with the adjacent non-tumor tissues (*P* = 0.045 and 0.006, respectively, Figure 2). Moreover, results demonstrated that the AHRR isoform without exon 8 was the predominant form either in tumor tissues (66.7%, 8/12) or in matched adjacent non-tumor tissues (100.0%, 12/12). There are 3 cases (number 1, 6, 8) which have higher total AHRR mRNA expression in tumor tissues compared with the adjacent non-tumor tissues, whereas, higher expression of AHRR isoform with exon 8 were determined in tumor tissues of case 6 and 8.

### AHRR Expression Analyzed by Western Blotting

**Figure 3:**
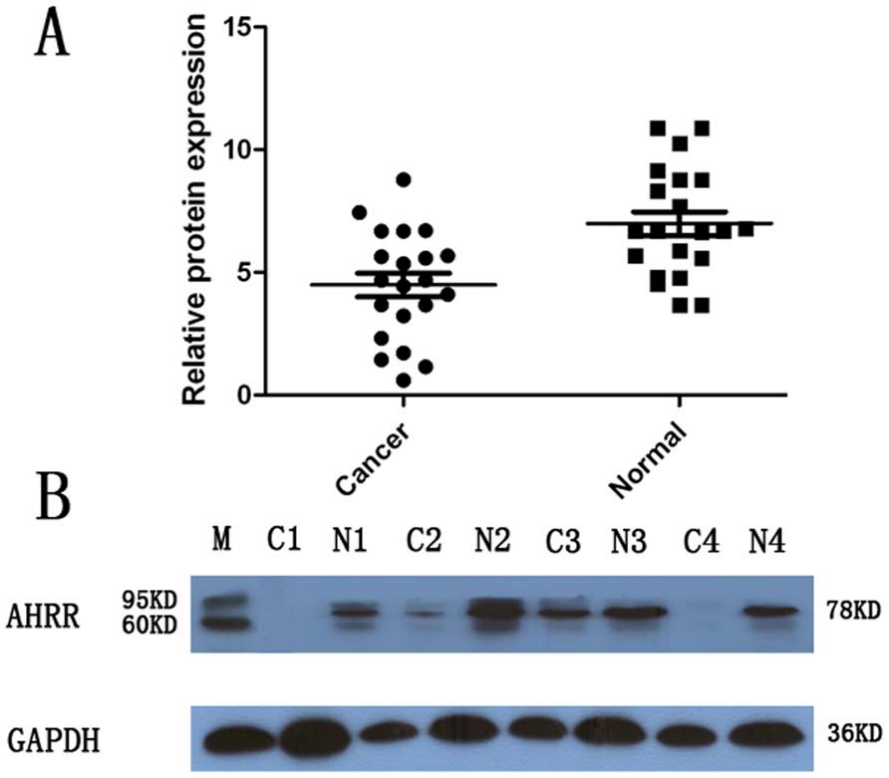
Decreased protein expression of AHRR in gastric cancer as assessed by western blotting. (A) Relative AHRR protein expression levels in gastric cancer tissues and noncancerous tissues (AHRR/GAPDH, n = 21, *P* = 0.004). The horizontal lines represent the means. (B) Representative result of AHRR protein expression in 4 paired gastric tumorous and matched adjacent nontumorous tissues (C, gastric cancer tissues; N, matched noncancerous gastric mucosa). M: molecular mass markers with the above band representing 95 kDa and the nether band representing 60 kDa.

The AHRR protein levels in the resected gastric cancer samples were determined by western blotting. The results showed an AHRR band at the expected size of 78 kDa, and the amount of AHRR protein present was further measured by densitometry. As shown in Figure 3, a decrease in AHRR expression was detected in 13 (61.9%) of the 21 tumor tissue samples, compared with the expression in the matched adjacent non-tumor tissue samples (*P* = 0.004, Figure 3A and Figure 3B). These findings were consistent with those of the qRT-PCR.

### The Association between Levels of AHRR Expression and Clinicopathological Characteristics, Based on Immunohistochemical Staining

**Figure 4:**
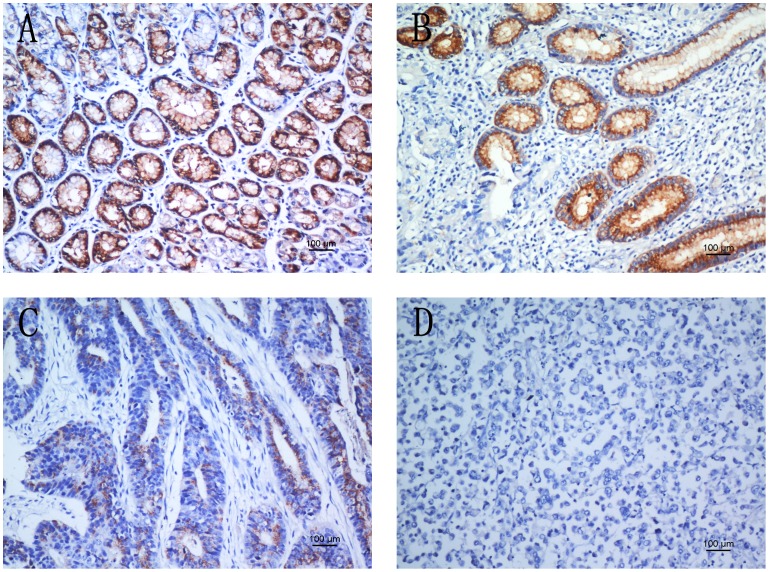
AHRR protein expression in gastric cancer surgical specimens shown by immunohistochemistry. (A) Strong AHRR staining was observed in noncancerous gastric mucosa. (B) Strong AHRR staining in well-differentiated gastric cancer. (C) Weak AHRR staining in moderately differentiated gastric cancer. (D) Negative AHRR staining in poorly differentiated gastric cancer.

To obtain further insight into the effect and prognostic value of AHRR expression in gastric cancer patients, paraffin-embedded tissue sections (n = 410) with histopathologically confirmed gastric adenocarcinoma were examined using immunohistochemistry. In the current study, AHRR expression was immunohistochemically localized to the cytoplasm. According to this present study, the AHRR immunoreactivity presented significant differences between the tumor tissue samples and the adjacent non-tumor ones. Overall, 235 cases (57.3%) showed positive AHRR expression in the tumor tissue samples, whereas the remaining 175 cases (42.7%) displayed reduced cytoplasmic AHRR expression (Table 1). Based on the categories defined with the aforementioned methods, the decreased expression of AHRR was significantly correlated with tumor size (*P*<0.001), depth of tumor infiltration (T stage, *P*<0.001), distant metastases (M) (*P* = 0.004) and TNM stage (*P*<0.001), but not with age, gender or local lymph node metastasis (N stage). Representative photomicrographs were shown in Figure 4.

**Table 1:** Correlation between AHRR expression and clinicopathological variables of 410 gastric cancer cases.

Clinicopathological parameters	*n* a	AHRR expression		?^2^	*P* value
		High	Low		
**All**	410	235	175		
**Age (years)**					
<55	179	109	70	1.661	0.227
≥55	231	126	105		
**Gender**				0.223	0.674
Male	270	157	113		
Female	140	78	62		
**Tumor size**				12.738	<0.001*
<3 cm	63	49	14		
≥3 cm	347	186	161		
**Tumor infiltration**				28.352	<0.001*
T1	47	39	8		
T2	38	30	8		
T3	42	20	22		
T4a	221	120	101		
T4b	62	26	36		
**Local lymph node metastasis**				5.691	0.129
N0	137	85	52		
N1	71	38	33		
N2	73	47	26		
N3	129	65	64		
**Distant metastasis**				8.846	0.004*
M0	366	219	147		
M1	44	16	28		
**TNM staging**				20.718	<0.001*
1	52	42	10		
2	142	83	59		
3	169	93	76		
4	47	17	30		

a Numbers of cases in each group.

* Statistically significant (*P*<0.05).

### Expression of AHRR and Clinical Outcome

**Figure 5:**
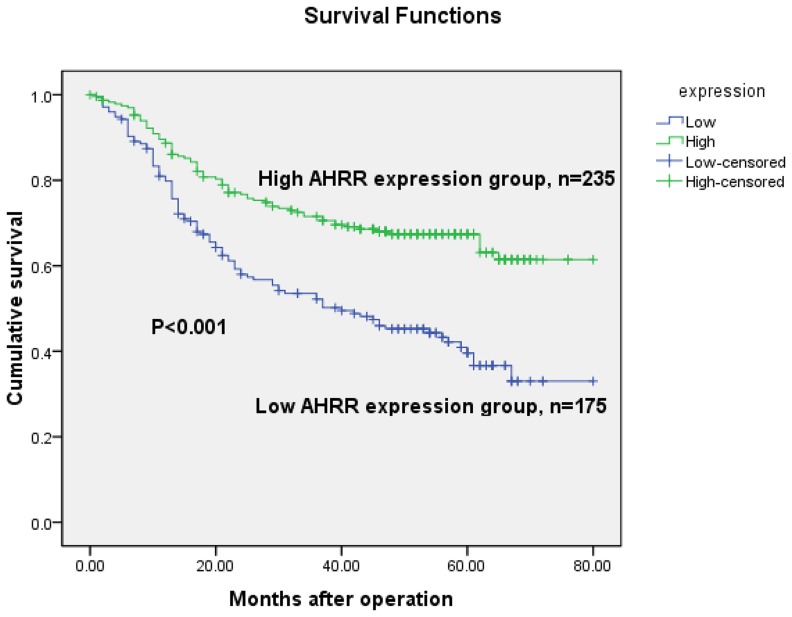
Kaplan-Meier survival curves of gastric cancer patients (n = 410) after gastrectomy. The survival rate of the patients in the AHRR-low group was significantly lower than that of the patients in the AHRR-high group (log-rank test, *P*<0.001).

The 5-year overall survival rates in patients with high and low AHRR expression were 69.4% and 45.7%, respectively. The overall survival of patients with low AHRR expression was significantly shorter than that of patients with high AHRR expression (*P*<0.001, log-rank test, Figure 5). Univariate Cox regression analyses showed that depth of tumor infiltration, local lymph node metastasis, distant metastasis, TNM stage, tumor size and AHRR expression were significantly interrelated with overall survival (Table 2). Furthermore, a multivariate Cox regression analysis confirmed distant metastasis (*P* = 0.044) and AHRR expression (*P* = 0.004) as independent predictors of the overall survival of patients with gastric adenocarcinoma (Table 2).

**Table 2:** Univariate and multivariate analyses of overall survival of gastric cancer patients.

Variables	*n* a	Univariate analyses			Multivariate analyses		
		HR	(95% CI)	*P* value	HR	(95% CI)	*P* value
**Age (years)**				0.181			
<55	179	1.000					
≥55	231	1.229	0.909–1.661				
**Gender**				0.947			
Female	140	1.000					
Male	270	0.990	0.727–1.346				
**Tumor size**				<0.001*			0.635
<3 cm	63	1.000			1.000		
≥3 cm	347	4.382	2.155–8.910		1.198	0.567–2.530	
**Tumor infiltration**				<0.001*			0.289
T1	47	1.000			1.000		
T2	38	6.601E3	0.000–1.045E34		9.683E3	0.000–9.369E44	
T3	42	2.527E4	0.000–3.987E34		1.243E4	0.000–1.201E45	
T4a	221	3.482E4	0.000–5.487E34		1.477E4	0.000–1.427E45	
T4b	62	7.151E4	0.000–1.127E35		2.067E4	0.000–1.997E45	
**Local lymph node metastasis**				<0.001*			0.223
N0	137	1.000			1.000		
N1	71	2.439	1.442–4.127		0.954	0.513–1.776	
N2	73	2.941	1.764–4.902		1.086	0.578–2.040	
N3	129	5.309	3.418–8.248		1.438	0.787–2.627	
**Distant metastasis**				<0.001*			0.044*
M0	366	1.000			1.000		
M1	44	5.379	3.731–7.756		3.835	1.036–14.191	
**TNM staging**				<0.001*			0.052
1	52	1.000			1.000		
2	142	14.990	2.055–109.348		3.789	0.444–32.355	
3	169	41.842	5.835–300.059		7.440	0.808–68.514	
4	47	121.343	16.657–883.966		5.403	0.401–72.805	
**AHRR**				<0.001*			0.004*
Low	175	1.000			1.000		
High	235	0.482	0.357–0.650		0.635	0.464–0.868	

HR, hazard ratio;

CI, confidence interval;

a Numbers of cases in each group;

* Statistically ignificant (*P*<0.05).

## Discussion

Tumor progression arises as a consequence of a series of cellular events, which involve but are not limited to deregulation of cell proliferation, resistance to apoptosis, enhanced cell motility, augmented angiogenic potential, and anomalies in cell-cell interaction and the microenvironment, resulting in tumor formation, invasion and metastasis [19]. In recent decades, we have verified that the process associated with tumor progression is regulated precisely by a small subset of genes that act by either enhancing (oncogenes) or diminishing (tumor suppressor genes) the final malignant outcome [20]. AHRR, as a member of the bHLH-PAS protein family, which was firstly discovered in 1999 [13], is a feedback inhibition modulator of the aryl hydrocarbon receptor (AHR) and exerts its effect by competing with AHR for aryl hydrocarbon receptor nuclear translocator (ARNT), thereby blocking AhR-dependent gene expression [21]. This feedback modulation plays a pivotal role in moderating AHR in oncogenesis and altered immune function [22].

Previously, statistics have shown a consistent downregulation of AHRR throughout many types of tumors, including colon, breast, lung, cervical, and ovarian cancer, when compared with normal tissues of the same anatomical origin [9]. Specifically, using tissue microarray and immunohistochemistry, it was found that intratumoral AHRR was inversely correlated with time to recurrence and overall survival of hepatocellular carcinoma patients after resection [16]. However, to date, the prognostic significance of AHRR in gastric adenocarcinoma has not yet been evaluated. In the current study, we estimated the expression of AHRR in gastric adenocarcinoma by real-time PCR, western blotting and immunohistochemistry, in addition to analyzing its clinicopathological and prognostic significance in a large amount of human samples. We illustrated that AHRR was expressed at lower levels of both mRNA and protein in gastric adenocarcinoma tissues than in corresponding non-cancerous mucosa, in agreement with previous statistics shown in other types of tumor samples [9].

Moreover, we determined relative mRNA expression of two AHRR isoforms (with or without exon 8) by qRT-PCR in 12 cases of paraffin-embedded tissues. Consistent with the total AHRR mRNA expression results, the AHRR isoform without exon 8 expression were significantly reduced in tumor tissues compared with the adjacent non-tumor tissues (*P* = 0.006). Besides, AHRR isoform without exon 8 was the predominant form either in tumor tissues (66.7%, 8/12) or in matched adjacent non-tumor tissues (100.0%, 12/12). AHRR gene has been reported to encode two isoforms and the isoform without exon 8 is the active isoform, which is the predominant form of AHRR expressed in multiple human tissues and human tumor cell lines [15], [23], [24]. Our results indicated that the predominant and active form of AHRR in gastric cancer may be the isoform without exon 8, which was consistent with the study by Karchner *et al* [15]. However, the functional role and mechanisms of active AHRR isoform in gastric cancer are unclear, which needs further investigation in the future research.

As a transcription factor, AHRR localizes initially in cytoplasm, interacts with ARNT and translocates to nucleus for prominent localization [13]. Mimura *et al* reported that AHRR was found to be localized in the nuclei [13]. However, in the current study, we observed exclusively a cytoplasmic expression pattern of AHRR proteins in gastric adenocarcinoma tissues. As was reported by previous researches, some other transcription factors have been described to be both cytoplasmic and nuclear expression in many kinds of human malignancies [25]–[27]. For the reason of these differences, Wang W *et al* supposed that immunohistochemistry may only evaluate the end products of gene expression [27]. We speculated that some methodological factors, such as tissue processing, heterogeneity of different kind of malignancies and antigen specificity may contribute to the differences. Besides, modifications of AHRR or changes of AHRR protein itself may also interfere with these results. Our results showed significantly decreased expression of AHRR in gastric cancer tissues, and confirmed the expression of AHRR as an independent risk factor for primary gastric adenocarcinoma patients.

Nevertheless, recent data are beginning to shed light on what may be a critical role, or the molecular mechanism, for the AHRR in cancer. For example, in human mammary tumor cell lines, AHRR knock-down with siRNA enhances AHR activity, confirming the assumption that AHRR constitutively represses AHR activity in tumors [28]. Interestingly, murine breast tumors induced with DMBA generally express extremely high AHR levels [29]. Moreover, as was reported by Hahn and collaborators, human tumors with relatively high levels of AHR apt to express low AHRR levels, strongly suggesting that AHR may suppress AHRR transcription and thereby to maximize its own activity [28]. With mutation detection, single-stranded polymorphism analysis, methylation specific PCR and gene function testing, Enrique Zudaire *et al* uncovered high rates of LOH and hypermethylation in the promoter region of the AHRR gene in colon, breast and hepatocellular carcinoma [18]. These previous data consistently supported the assumption that AHRR plays as a tumor suppressor gene in several types of human cancer. Recently, Haarmann-Stemmann T’s study proposed that AHRR expression in tumor cells inversely correlates with their angiogenic potential. Tumor cells with over expressed AHRR presented lower angiogenic potential, whereas tumor cells in which AHRR expression was blocked showed high angiogenic potential [21].

Our observations are consistent with the idea that AHRR plays as a tumor suppressor and further suggest that AHRR might play an important role in the tumor progression of gastric cancer. Furthermore, in our study, which encompassed a relatively large number of gastric cancer patients (n = 410), we supported the hypothesis that AHRR acts as a tumor suppressor in gastric adenocarcinoma because low AHRR expression was associated with tumors with depth of tumor infiltration (T) (*P*<0.001), distant metastases (M) (*P* = 0.004) and TNM stage (*P*<0.001). Consistent with our findings, decreased AHRR expression was reported to be significantly associated with a higher grade of hepatocellular cancer [16]. A Kaplan-Meier survival analysis showed a significant correlation between low expression of AHRR and poorer clinical outcome of gastric cancer patients after radical operation. Cox hazard ratio regression analyses further demonstrated that the AHRR expression level was an independent risk factor for overall survival, suggesting that this value may serve as a prognostic biomarker for gastric cancer patients after surgery. These data suggest that examination of AHRR expression might be helpful in guiding clinical management. However, the functional role and mechanisms of AHRR in gastric cancer are unclear, which needs further investigation in the future research.

In conclusion, the present study suggests that low AHRR expression independently predicts worse overall survival in patients with gastric adenocarcinoma. However, the molecular mechanisms involved in the regulation of AHRR in gastric cancer require further investigation. Future studies in this field are necessary because a better understanding of AHRR function in malignancies has the potential to improve the prognosis of gastric cancer. Moreover, we expect that AHRR may function as a useful target for new therapeutic interventions against gastric adenocarcinoma.

## Materials and Methods

### Ethics Statement

The research was approved by the Ethics Committee of Sun Yat-sen University Cancer Center, and written informed consent was obtained from each patient involved in the study.

### Patients

From January 2000 to December 2006, clinicopathological data from 410 gastric cancer patients who underwent surgical resection at Sun Yat-sen University Cancer Center were retrospectively analyzed. Patients who met the following eligibility criteria were included: (1) diagnosis of gastric adenocarcinoma identified by histopathological examination; (2) surgical history that included gastrectomy plus lymphadenectomy (limited or extended); (3) availability of complete follow-up data; (4) no preoperative treatment, such as chemotherapy and radiotherapy; (5) no history of familial malignancy or other synchronous malignancy (such as GIST, esophageal cancer, or colorectal cancer); (6) no recurrent gastric cancer or remnant gastric cancer; and (7) no death in the perioperative period. Tumor resection and D2 lymphadenectomy were performed by experienced surgeons, and the surgical procedures, which followed the Japanese Gastric Cancer Association (JGCA) guidelines [23], were similar in all patients who underwent radical resections.

Fresh gastric cancer and adjacent non-tumor tissue samples (n = 40) were obtained from 40 gastric cancer patients who underwent surgical resection at the Sun Yat-sen University Cancer Center between 2009 and 2011. After surgical resection, the fresh tissue samples were immediately immersed in RNAlater (Ambion, Inc., USA) and stored at 4°C overnight to allow thorough penetration of the tissues; the samples were then frozen at −80°C until RNA extraction. Both the tumor tissue and the adjacent non-tumor tissue, which was located more than 2 cm away from the gastric cancer, were sampled and then verified by pathological examination. Paraffin-embedded samples were obtained from the 410 gastric cancer patients who underwent surgical resection at the Sun Yat-sen University Cancer Center between 2000 and 2006. Paraffin-embedded tissues of 12 cases used to determine the expression of two AHRR isoforms were obtained from the 410 gastric cancer patients related above. Each tumor sample was assigned a histological grade based on the World Health Organization (WHO) classification criteria. All of the patients were staged using the 7th edition of the International Union Against Cancer (UICC) Tumor-Node-Metastasis (TNM) staging system.

### Extraction of Total RNA and Real-time Quantitative PCR

The total RNA was extracted using TRIzol (Invitrogen, Carlsbad, California, USA) according to the manufacturer’s protocol. The total RNA of the 12 Paraffin-embedded tissues used to determine expression of the two AHRR isoforms was extracted using the Allprep DNA/RNA FFPE kit (QIAGEN, Germany) according to the manufacturer’s protocol. The total RNA concentration was assessed by measuring the absorbance at 260 nm using a NANO DROP spectrophotometer (ND-1000, Thermo Scientific, USA). Reverse transcription (RT) to synthesize the first-strand of cDNA was performed with 2 µg of total RNA treated with M-MLV reverse transcriptase (Promega, USA) according to the manufacturer’s recommendations. The resulting cDNA was then subjected to real-time quantitative PCR for evaluation of the relative mRNA levels of AHRR and GAPDH (as an internal control) with the following primers: AHRR forward, 5′-CTTAATGGCTTTGCTCTGGTCG-3′, and reverse, 5′-TGCATTACATCCGTCTGATGGA-3′; AHRR isoform with exon 8 froward, 5′-TCTGCTGTCCCGAGCCACT-3′, and reverse, 5′-TGCTGCTCCTTCCTGCTGA-3′; GAPDH forward, 5′-CTCCTCCTGTTCGACAGTCAGC-3′, and reverse: 5′-CCCAATACGACCAAATCCGTT-3′. Gene-specific amplification was performed using an ABI 7900HT real-time PCR system (Life Technologies, Carlsbad, California, USA) with a 15 µl PCR mix containing 0.5 µl of cDNA, 7.5 µl of 2×SYBR Green master mix (Invitrogen, Carlsbad, California, USA), and 200 nM of the appropriate oligonucleotide primers. The mix was preheated at 95°C (10 min) and then amplified at 95°C (30 sec) and 60°C (1 min) for 45 cycles. The resolution curve was measured at 95°C for 15 sec, 60°C for 15 sec and 95°C for 15 sec. The Ct (threshold cycle) value of each sample was calculated from the threshold cycles with the instrument’s software (SDS 2.3), and the relative expression of total AHRR mRNA and mRNA levels of both isoforms was normalized to the GAPDH value. The data were analyzed using the comparative threshold cycle (2^−ΔCT^) method, and the relative mRNA level of AHRR isoform without exon 8 was analyzed using the total mRNA level subtracting the mRNA level of AHRR isoform with exon 8.

### Western Blotting Analysis

The frozen tissue samples from patients with gastric cancer, including the tumor and non-tumor tissue, were homogenized in RIPA lysis buffer, and the lysates were cleared by centrifugation (12,000 rpm) at 4°C for 15 min. Approximately 40-mg protein samples were run on a 12% SDS-PAGE gel and were transferred to PVDF membranes. After blocking non-specific binding sites for 60 min with 5% non-fat milk, the membranes were incubated overnight at 4°C with a primary polyclonal antibody against AHRR (Abcam, USA, at a 1∶1000 dilution). The membranes were then washed three times with TBST for 10 min each and probed with an HRP conjugated secondary antibody (Immunology Consultants Laboratory, USA, at a 1∶2000 dilution) for 60 min at room temperature. The membranes were then washed three times with TBST and developed with an enhanced chemiluminescence system (ECL, Pierce). The molecular mass markers (Jetway Biotech, China) were processed as described above. The protein levels were normalized to that of GAPDH detected using HRP conjugated primary anti-GAPDH antibody (Medical & Biological Laboratories, Japan, at a 1∶10000 dilution) that can be developed by the enhanced chemiluminescence system without incubated with secondary antibody.

### Immunohistochemistry Analysis

The tissue sections were deparaffinized with dimethylbenzene and rehydrated through 100%, 95%, 90%, 80% and 70% ethanol. After three washes in phosphate-buffered saline (PBS), the slides were boiled in antigen retrieval buffer containing 0.01 M sodium citrate-hydrochloric acid (pH = 6.0) for 15 min in a microwave oven. After rinsing with PBS, the tissue sections were incubated with primary antibody, and the slides were then rinsed in 3% peroxidase quenching solution (Invitrogen) to block endogenous peroxidase. The sections were then incubated with a mouse monoclonal antibody against AHRR (Abcam, USA, at a 1∶500 dilution) at 4°C overnight and then incubated with horseradish peroxidase (HRP) (ChemMateTM DAKO EnVisionTM Detection Kit) at room temperature for 30 min. After washing in PBS, the visualization signal was developed with 3, 3'-diaminobenzidine (DAB) solution, and all of the slides were counterstained with hematoxylin. As negative controls, adjacent sections were processed as described above, except that they were incubated overnight at 4°C in blocking solution without the primary antibody.

The total AHRR immunostaining score was calculated as the sum of the percentage of positively stained tumor cells and the staining intensity. Briefly, the percentage of positive staining was scored as 0 (0–9%, negative), 1 (10%–25%, sporadic), 2 (26%–50%, focal) or 3 (51%–100%, diffuse), and the intensity as 0 (no staining), 1 (weak staining), 2 (moderate staining) or 3 (dark staining). The total immunostaining score was calculated as the value of percent positivity score × staining intensity score, which ranged from 0 to 9. The expression level of AHRR was defined as following: “−” (negative, score 0), “+” (weakly positive, score 1–3), “++” (positive, score 4–6), “+++” (strongly positive, score7–9). We defined AHRR high expression as a total score ≥3, and low expression as a total score <3.

### Statistical Analysis

Differences in mRNA and protein expression between tumor samples and the paired adjacent non-tumor tissue samples were evaluated with the paired-samples t-test. The χ^2^ test was used to analyze the relationships between AHRR expression and various clinicopathological parameters. Survival curves were calculated using the Kaplan–Meier method and compared by the log-rank test. The Cox proportional hazard regression model was used for univariate and multivariate analyses to study the effects of the clinicopathological variables and AHRR expression on survival. The statistical analyses were performed with the Statistical Package for the Social Sciences, version 17.0 (SPSS Inc., Chicago, IL, USA), and a two-sided P value less than 0.05 was considered to be statistically significant.
